# Optimizing a Text Message Intervention to Reduce Heavy Drinking in Young Adults: Focus Group Findings

**DOI:** 10.2196/mhealth.5330

**Published:** 2016-06-22

**Authors:** Brian Suffoletto, Jeffrey Kristan, Laurel Person Mecca, Tammy Chung, Duncan B Clark

**Affiliations:** ^1^ University of Pittsburgh Department of Emergency Medicine Pittsburgh, PA United States; ^2^ Qualitative Data Analysis Program University Center for Social and Urban Research University of Pittsburgh Pittsburgh, PA United States; ^3^ University of Pittsburgh Department of Psychiatry Pittsburgh, PA United States

**Keywords:** alcohol, young adult, text messages, qualitative

## Abstract

**Background:**

Recent trial results show that an interactive short message service (SMS) text message intervention, Texting to Reduce Alcohol Consumption (TRAC), is effective in reducing heavy drinking in non-treatment-seeking young adults, but may not be optimized.

**Objective:**

To assess the usability of the TRAC intervention among young adults in an effort to optimize future intervention design.

**Methods:**

We conducted five focus groups with 18 young adults, aged 18-25 years, who had a history of heavy drinking and had been randomized to 12 weeks of the TRAC intervention as part of a clinical trial. A trained moderator followed a semistructured interview guide. Focus groups were audiotaped, transcribed, and analyzed to identify themes.

**Results:**

We identified four themes regarding user experiences with the TRAC intervention: (1) ease of use, (2) comfort and confidentiality, (3) increased awareness of drinking behavior, and (4) accountability for drinking behavior. Participants’ comments supported the existing features of the TRAC intervention, as well as the addition of other features to increase personalization and continuing engagement with the intervention.

**Conclusions:**

Young adults perceived the TRAC intervention as a useful way to help them reduce heavy drinking on weekends. Components that promote ease of use, ensure confidentiality, increase awareness of alcohol consumption, and increase accountability were seen as important.

## Introduction

Heavy episodic drinking, typically defined as consuming four or more drinks for women and five or more drinks for men over a drinking occasion, is the most common pattern of excessive alcohol consumption in the United States [1]. Young adults have an especially high prevalence of heavy episodic drinking [2] and suffer a multitude of related health and social consequences including death and serious injury, primarily from motor vehicle accidents, homicides, and suicides [3]. Research studies have shown that brief counseling interventions can be effective in reducing alcohol consumption and related risks among young adults in various settings [4], but are limited in their ability to scale up due to costs and training requirements [5].

Computerized interventions may allow for economies of scale and standardization of procedures to ensure replicability that is less feasible with in-person interventions. As well, mobile communication technology allows computerized interventions to provide support over time in an individual’s natural environment, and to adapt feedback based on changing personal circumstances [6]. This may be especially useful for interventions targeting substance use [7], which is highly dependent on contextual challenges to self-regulation [8] and requires ongoing self-management [9].

The Texting to Reduce Alcohol Consumption (TRAC) intervention was iteratively developed from a systematic literature review of alcohol prevention interventions and short message service (SMS) text message interventions for health behaviors, and pilot studies [10-13]. Specifically, in 2010, we began developing the TRAC intervention, a computerized intervention using text messaging, to help young adults reduce alcohol consumption. In 2011, we completed a pilot randomized controlled trial (RCT) where we found that young adults used the TRAC intervention at high rates over a 12-week period and that it was potentially useful in helping them reduce heavy drinking [10]. In 2012, we conducted an online crowdsourcing study where young adults—78% with past hazardous drinking—ranked our existing text messages and created new messages they would find useful for reducing alcohol use [11]. In 2013, we used feedback from these prior studies to design an updated TRAC intervention, which is detailed in the Methods section. In 2014, we completed a randomized controlled trial of 765 young adults in which the TRAC intervention produced reductions in heavy drinking days compared to control and assessment-only groups out to 9-month follow-up [12,13]. Despite these findings, we found increasing nonadherence to the TRAC intervention over the 12-week intervention and a significant proportion of participants who continued to exhibit heavy drinking at follow-ups. This suggests a need to continue to improve the intervention’s usability, defined as the measure of the ease with which it can be learned and used, including its effectiveness and efficiency [14].

In this study, we address the need to continually improve the intervention through an iterative process of testing and evaluation, by collecting feedback from young adult drinkers who were exposed to the TRAC intervention. This iterative process follows the guiding principles of user-centered design [15] as well as recommendations for development of technology-based behavioral interventions [16]. This study is unique relative to existing literature on the formative development of text message interventions for alcohol use prevention [17-20]. It is unique in that we examine the opinions of individuals who actually experienced an intervention—as opposed to being presented with theoretical or sample messages—and who reflected on how the intervention’s various features impacted their thoughts, beliefs, and behaviors.

## Methods

### Study Design

The specific aim of this study was to understand young adults’ qualitative experiences using the TRAC intervention and explore ways to improve its usability. Five focus groups, each consisting of 3-5 people, were conducted. We probed user reactions to the TRAC intervention in general as well as specific TRAC intervention components, including drinking assessments, goal-setting prompts, and feedback messages. Finally, we gauged participant opinions about candidate additions to the TRAC intervention and probed for any other suggested changes. All participants provided written informed consent. Study procedures were approved by the Institutional Review Board at the University of Pittsburgh. None of the findings reported in this manuscript have been described in prior publications.

### Recruitment and Participants

**Figure 1:**
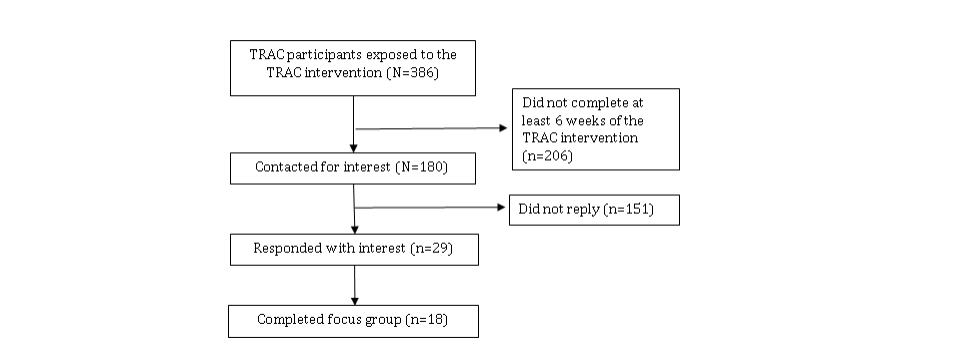
Flow diagram of focus group participants. TRAC: Texting to Reduce Alcohol Consumption.

We recruited focus group participants from those who met eligibility criteria for the TRAC trial (ClinicalTrials.gov number: NCT 01688245). TRAC participants were identified in four emergency departments (EDs) in Pittsburgh, Pennsylvania. TRAC trial inclusion criteria were as follows: 18-25 years of age, having screened positive for past hazardous alcohol consumption—Alcohol Use Disorders Identification Test Consumption (AUDIT-C) score >3 for women and >4 for men [21]—and not seeking help for their alcohol use. Inclusion for this focus group study also required randomization to the TRAC intervention condition (n=386). We limited focus group enrollment to those individuals who had completed at least 6 weeks (50%) of the SMS text messaging assessments so that participants would be able to reflect on actual experiences using the TRAC intervention. Invitations were sent via email to 180 individuals and we received preliminary interest from 29 (16.1%). For each session, we attempted to invite an equal number of women and men as well as at least one person who had self-identified as black. In total, 18 individuals took part in the focus group study. The flow diagram of focus group participants can be seen in Figure 1.

### The Texting to Reduce Alcohol Consumption Intervention

The overall goal of the TRAC intervention is to help young adults reduce their alcohol consumption. TRAC targets heavy episodic drinking, given its association with alcohol-related harms [3], and in particular, heavy episodic drinking on weekends because that is when most binge drinking occurs among young adults [22]. The design was chiefly informed by the theory of planned behavior [23] and goal-setting theory [24], incorporating self-monitoring, goal-commitment prompts, and immediate tailored feedback messages, all of which have been shown to be effective components of mobile behavioral interventions for reducing alcohol consumption [25]. The TRAC intervention was designed to function for individuals with a variety of levels of readiness to change, to accommodate shifting motivations from week to week, and to offer an action plan to anyone reporting likelihood of having a heavy drinking episode over the weekend. It was programmed to run for 12 weeks, a period that would provide time for individuals to learn to adopt safer drinking habits without repetition of the intervention. All content and user flow of the TRAC intervention was developed primarily by the first author (BS) in consultation with an adolescent addiction psychiatrist (DBC) and computer programmer. The program was run from servers and a modem pool housed at the university.

Upon enrollment to the TRAC intervention, participants received a series of welcome messages instructing them on what to expect over the 12 weeks and how to quit the program. The flow of text message branching logic can be seen in Multimedia Appendix 1 and a full description of the TRAC intervention with sample message libraries can be found in prior publications [12]. In brief, each Thursday at 6pm, participants were asked to report if they planned on drinking that weekend, and if they responded in the negative (ie, “no,” “nah,” or “N”), they received a message reinforcing their choice not to drink. If they responded that they planned on drinking (ie, “yes,” “yup,” or “Y”), they were asked whether they were likely to drink five or more drinks (for men) or four or more drinks (for women) on any occasion over the weekend. If they responded that they were not likely to have a heavy drinking episode over the weekend, they received a message encouraging their choice and another informing them about safe drinking. If they responded that they were likely to have a heavy drinking episode, they were asked whether they were willing to set a goal to limit their drinking to below the heavy drinking threshold—five or more drinks for men/four or more drinks for women— that weekend. If they responded that they were not willing to set a goal to limit their drinking, they received a message expressing understanding of the difficulty in making changes. If they responded that they were willing to set a goal to limit their drinking, they received a message encouraging that goal and another message encouraging use of a specific protective behavioral strategy.

Each Sunday at 12pm, participants were asked to report the most alcoholic drinks they had consumed on any single occasion between Thursday and Sunday. If they reported that they did not drink (ie, “none,” “zero,” or “0”), they received a message congratulating them for not drinking. If they reported a value greater than zero, but less than the heavy drinking threshold, they received a message acknowledging their report of drinking and another message with general alcohol education. If they reported drinking above the heavy drinking threshold, they received a message expressing concern and another message prompting them to reconsider their drinking behavior. All feedback messages were modeled on existing language used in brief interventions for alcohol use and in the spirit of motivational interviewing.

### Focus Groups

We chose to conduct focus groups for several reasons. First, focus groups are a fast and efficient method for obtaining data from multiple participants [26]. Second, focus groups can increase participants’ sense of belonging [27] and help them to feel safe, facilitating sharing of information [28]. This may be particularly relevant to sensitive topics such as alcohol use [29]. Third, focus groups can stimulate conversations around ideas or themes, yielding important data not elicited in one-on-one interviews [30].

Before conducting any focus groups, a standardized, semistructured qualitative guide was developed to increase consistency across interview sessions. We pilot-tested the interview guide among 3 young adult volunteers—2 female and 1 male— not previously exposed to the TRAC intervention and made refinements based on their feedback. The guide probed user reactions to the TRAC intervention in general as well as to the three main features of the TRAC intervention: drinking plan and consumption queries, goal-setting prompts, and feedback messages. The interview guide also probed opinions about possible additions to the TRAC intervention. Specifically, we were interested in understanding how young adults felt about incorporating text messaging during drinking episodes because of prior trial findings that a significant proportion of young adults who set drinking limit goals still did not meet them, and our belief that messages delivered more proximal to actual drinking events could enhance potency. We also probed opinions about a Web-based dashboard as an adjunct to text messaging given prior expressed desire among young adult participants for an easier way to track their progress over time.

Each focus group was scheduled to comprise small groups—3-4 individuals—to ensure participant interaction and comfort [31]. Each focus group lasted about 90 minutes and was conducted in a private university conference room in the evening. The focus groups were conducted by a female facilitator (LPM) with expertise in focus group techniques and qualitative methodology.

Before each focus group began, participants provided written informed consent. The consent highlighted the potentially sensitive nature of the discussions and urged participants to not share discussion content outside the group. As well, at the beginning of each focus group, the moderator stressed the confidential nature of the discussion to the participants. Participants were told that the focus group discussion would be audiotaped, would be used for research purposes only, and would not be accessible to anyone outside the research team. To ensure confidentiality, participants were told not to use their full names. It was stressed that the opinions of the participants were important and that there were no right or wrong answers.

The focus groups started with a warm-up where participants were reminded of the TRAC intervention format. Probes were used to encourage clarification and evoke greater detail from participants’ narratives. A research assistant took detailed field notes during the focus groups and the groups were audiotaped and transcribed by personnel at the Qualitative Data Analysis Program at the University Center for Social and Urban Research at the University of Pittsburgh. At the conclusion of each focus group, we debriefed all participants on the preliminary results of the randomized trial and asked for any closing remarks or questions. When all participants’ questions had been exhaustively answered, the participants were thanked and provided with a debit card worth US $30.

### Data Analysis

We chose a thematic content analysis approach and used the qualitative research software package, ATLAS.ti 5.0 (Scientific Software Development). A preliminary codebook was created based on close readings of the first transcripts, incorporating explicit domains from interview guides (deductive themes) as well as recurrent unanticipated themes that were emergent across transcripts (inductive themes). Provisional definitions were given to each code and two analysts applied the codes to each transcript. The coded transcripts were merged for comparison and code definitions were revised based on an examination of coding disagreement. Coded text was further reviewed through an iterative process, resulting in refined themes. We did not record which individual participant said which statement or count how many participants agreed or disagreed with a given statement. In presenting the results, we chose participant quotes that represented both the majority sentiments within each theme as well as any quote that offered a contrasting opinion within that theme.

## Results

### Overview

We conducted five focus groups with 18 participants—12 females (67%) and 6 males (33%). Overall, the sample was 44% (8/18) non-Hispanic black and 50% (9/18) non-college educated; 28% (5/18) were under the US legal age of 21 years to purchase or consume alcohol. There was a high rate of cannabis use (11/18, 61%) and daily tobacco use (7/18, 39%) among our cohort. Other baseline characteristics of focus group participants can be seen in Table 1. During the trial, focus group participants responded to an average of 97% of Thursday text message queries and 95% of Sunday text message queries.

**Table 1:** Baseline characteristics of focus group participants (n=18).

Characteristics		Mean (SD) or n (%)
Age in years, mean (SD)		22 (2)
Underage (<21 years), n (%)		5 (28)
Female, n (%)		12 (67)
**Race, n (%)**		
	Black	8 (44)
	White	10 (56)
	Other	0 (0)
	Hispanic	0 (0)
College educated, n (%)		9 (50)
**Employment, n (%)**		
	Not working	5 (28)
	Part time	5 (28)
	Full time	8 (44)
**Other substance use** **(past 3 months), n (%)**		
	Tobacco, daily or almost daily	7 (39)
	Any cannabis	11 (61)
AUDIT-C^a^ score, mean (SD)		7 (2)

^a^AUDIT-C: Alcohol Use Disorders Identification Test Consumption.

### Overall Themes

Analysis revealed the following four major themes regarding experience with the TRAC intervention: (1) ease of use, (2) comfort and confidentiality, (3) increased awareness of drinking behavior, and (4) accountability for drinking behavior.

#### Theme One: Ease of Use

Participants expressed that text messaging was an ideal electronic modality to deliver drinking support. They naturally contrasted text messaging with other electronic modalities. For example, one participant explained, “I remember thinking it was a great method because it’s—you can respond to it very quickly, it’s not like you have to log into a computer.” Another said, “It’s convenient, especially for us for our generation, I think more so than being on the computer, you know, checking our emails.” Another described, “It’s way better than the app because it hits you. You know, it’s a text message right straight to your phone.” One participant did however comment that text messaging is easy to use but may not be the most impactful modality given that it is limited to text and 160 characters: “Even though it is very accessible, I don’t know if it’s the most, you know, high impact way to reach somebody.”

#### Theme Two: Comfort and Confidentiality

Overall, participants seemed to feel comfortable texting about their drinking behaviors. In fact, they described how the text messaging modality was preferred to in-person disclosure of drinking, with a resultant feeling of not being judged. For example, one participant said, “I thought it was more comfortable just because when you’re in front of somebody and you’re like, ‘Oh, I had 20 drinks.’ Some people are kind of like, ‘20 drinks?! That’s a lot.’” Regarding confidentiality, one participant said, “I felt like nobody was going to see it. I know someone was going to see it but it was like, it was on [my] phone, it’s gone, I don’t have to worry about it again, you know? And if I don’t want anyone to look at my phone and see that, then I just delete it.” Some participants did express some concerns about confidentiality, but that it became less of a concern as the intervention continued. One participant summed up his evolving feelings this way: “I thought it was kind of funny at first just like I’m texting this nobody, I didn’t know if it was real or something...but it just became routine...and then I was like, ‘You know what? I’m being honest now.’”

#### Theme Three: Awareness of Drinking Behavior

Participants indicated that they were not very aware of the extent of their drinking prior to starting the TRAC intervention. For example, one participant said, “I really went into it thinking like, ‘I’m not really going to need this because I don’t drink too much.’” Many recalled that the repetitive nature of the week-to-week drinking assessments helped them develop a habit of paying attention to how much they drank. For example, one participant said, “The consistency keeps reminding you...then you started to think, ‘Well, am I drinking this much this weekend?’” Several participants commented on how it is difficult to estimate drinking quantity in the real world. For example, one participant said, “You know, I might have had four red solo cup glasses, but you know there could have been two shots in there, could have been three shots in there.”

Participants commented that the feedback messages on Sunday, after reporting weekend drinking quantity, made them more aware of their drinking, but individuals seemed to have differing opinions on which types of feedback messages were preferred. Some participants liked the statistics on the dangers of heavy drinking: “Any time I get statistics, they obviously like stick in people’s heads with cigarette smoking, drinking, STD, like anything. So, I love the statistics.” Others felt that the messages were not telling them anything they did not already know. For example, one said, “I remember thinking, ‘[pfft] okay, whatever.’ And even with the [protective behavioral] strategies, I think we know what we’re supposed to be doing.”

Many discussed how the TRAC intervention helped them become aware of the drinking habits of their peers. For example, one participant commented, “When I go to parties or I see people drinking, I’m just like, ‘Do you know how many drinks you’re consuming?’ Like I count people’s drinks for them...and I’m like, ‘Why are you doing that to yourself?’” Another participant commented about how the intervention influenced their peer group: “It helped my friends too because they’d see me doing [TRAC] and they’d be like, ‘Oh, maybe we shouldn’t have like 12 shots before we go out.’”

#### Theme Four: Accountability for Drinking Behavior

Overall, participants seemed to feel that the TRAC intervention made them feel more accountable for their drinking choices. One participant illustrated this point in saying, “The fact that it’s constantly in your face and you get a chance to see how much you drink and how often it is, makes you—makes your mind wonder. And it helps you to kind of be able to control it [your drinking], if you want to.” Participants felt that the one feature that seemed to bring about the feeling of accountability was the goal-commitment query sent each Thursday, commenting that it made them be more mindful when they were out drinking. For example, one participant said, “...I thought about it [the goal] when I don’t think I normally would have. So, even if I didn’t reach it [low-drinking-quantity goal], I was at least thinking about the amount I was drinking, which was a positive.” Another TRAC intervention feature that seemed to bring about the feeling of accountability was the alcohol consumption recall query sent each Sunday. One participant recalled, “I’d wake up, you know, ridiculously hungover day after day and I’d start to see these messages. I’m like, ‘Alright, well I should kind of like tone it back.’”

Some participants expressed some discomfort with the feeling of being held accountable. For example, one participant said, “It’s like your mom telling you, ‘What the heck are you doing?’ but in a text. But it made you think about it, and sometimes, yeah, it was uncomfortable, but if you’re honest about it, then it’s gonna help you recognize you have a problem.” Some mentioned that the feeling of being accountable for their drinking lasted beyond the intervention period. For example, “I still count. Like I still—I don’t have a calendar but I keep track, which I never did because I never thought about it.”

### Suggestions for Improvement of the Texting to Reduce Alcohol Consumption Intervention

Discussion revealed areas for improvement. For example, some participants reported intervention fatigue; one participant stated, “Sometimes I would answer honestly, but if I didn’t really feel like all the texting, I would just be like, ‘I followed the goal, followed through with my goal’ just so I wouldn’t have to keep going.” However, at least one participant suggested providing the option of continuing the program for longer than 12 weeks, if desired.

Participants had noteworthy suggestions for reducing intervention fatigue and improving engagement with the TRAC intervention. In general, participants supported the use of more personalization by sending messages using their first names. Participants also suggested incorporating more tailored feedback. For example, one participant said, “Maybe if you could identify the factor or reason why they were trying to quit, so like saving money and you factor that into the messages.” Regarding tailoring of the goal-commitment query, participants suggested using drinking quantity thresholds higher than the four or five drinks per occasion recommended by the National Institute on Alcohol Abuse and Alcoholism (NIAAA). One participant commented that the recommended maximum drinking quantities during Thursday goal-setting prompts were “pretty much nothing“—that is, they were too low to experience an effect from drinking.

When we probed the possibility of incorporating text messages to support low-quantity drinking goals during drinking episodes, participants were ambivalent. For example, one participant stated, “I mean everybody is on their phone when they’re drinking, but I think that some other people might get offended or might get annoyed, irritated. But I think for me, that would be beneficial, just as kind of a reminder in the back of your head, ‘Hey, just reminding you, be mindful of how much you’re drinking.’” A participant suggested that one-way “push” messages, which provided a reminder of the goal to limit drinking, could help to optimize the effect of goal setting while minimizing participant burden.

When we probed the possibility of incorporating a Web-based dashboard where their text message data could be stored and graphically displayed, participants were generally supportive. One suggested addition was the ability to compare progress to others. For example, one participant said, “I like to see things quantified and it would actually be interesting, like same age, peers. This is their trend.” It was suggested that the dashboard also incorporate some form of gamification. For example, one participant said, “I could see a lot of value in some sort of a reward scheme.” Additionally, it was suggested that there be a social network component. One participant illustrated how this would be helpful: “If you just had maybe like a website that, you know, just had...other people’s stories and experiences. Like I don’t know how many people would be like, ‘Oh my gosh, my weekend was terrible, anybody else feel the same way?’”

## Discussion

### Principal Findings

This focus group study summarizes the opinions of non-treatment-seeking young adults with past hazardous alcohol consumption who were exposed to an interactive 12-week text message intervention focused on reducing weekend heavy drinking episodes. Participants recalled positive experiences with the TRAC intervention, citing ease of use and immediacy in the natural context of life as major facilitators in reducing alcohol use. Participants recounted how the intervention made them more aware about their own drinking patterns and felt it prompted accountability to drink more moderately. Although each intervention piece, namely the weekend drinking plan query, the goal-commitment prompt, and the Sunday alcohol consumption check-in, seemed to play unique roles, the intervention pieces also seemed to function together synergistically. Moreover, the repetition from week to week seemed to help individuals build alliance with the intervention over time and support reduction in drinking behavior.

Participant comments regarding the ease of use is not surprising given that text messaging is ubiquitous on mobile phones, a common mode of communication among young adult drinkers, and combines immediacy with the ability to reply asynchronously [32]. Participant overall comfort with communicating about drinking and feelings of adequate confidentiality may be surprising to some given that text messaging is considered an “unsecure” communication modality. However, research suggests that communication about sensitive topics such as alcohol and other substance use may actually be preferred through electronic modalities [33].

The TRAC intervention seemed to help individuals learn how to track the amount of alcohol they consumed. Although the act of assisting self-monitoring of drinking through Sunday alcohol consumption check-ins may be an important mechanism of action, it is clearly not the only necessary element. For example, in our trial findings, the assessment-only control group, which received Sunday alcohol consumption check-ins but did not receive feedback, showed no reductions in alcohol consumption at follow-ups [13]. One possible explanation is that self-monitoring of drinking quantity alone, without feedback, is not enough to change behavior among young adult drinkers, as has been found in other studies of alcohol use [34]. Another possible explanation may be that *awareness* of alcohol consumption occurs as a result of other intervention components (eg, goal-commitment prompts) or the components acting together as a whole.

The TRAC intervention elicited feelings of accountability in some individuals, which seemed to stem from developing a discrepancy between their perception of their drinking and what they had *learned* about their drinking behavior over the course of the intervention. It also seemed to stem from their growing awareness of the hazardous drinking patterns of their peer groups. We speculate that these perceived discrepancies motivated many to change their drinking over the course of intervention exposure and fits with one of the main theoretical underpinnings of the TRAC intervention, the theory of planned behavior, which stresses the importance of goal setting and perceived norms regarding alcohol use on alcohol consumption [35]. We also noted that what began as feelings of accountability to the TRAC intervention appeared to shift to feelings of accountability to themselves over the course of intervention exposure. This suggests that accountability for limiting drinking may become internalized through repetition over the course of the intervention for some young adults.

There were a number of design improvements that participants suggested, including increasing personalization and tailoring, such as adding the first name to texts and providing the option to continue the intervention for longer periods. Some participants seemed to desire more adaptive goal-commitment prompts. Specifically, some felt that limiting themselves to four or five drinks was too low of a threshold to commit to, especially when they are used to drinking 10 or more drinks on a weekend evening. This notion was reflected in our trial, where we found that young adults were not willing to commit to a drinking limit goal around half the time. It also raises an interesting dilemma for operationalizing design, where we would essentially ask young adults to commit to drinking less than what they are used to but higher than four or five drinks, the current upper limit of what the NIAAA considers a moderate level of alcohol use. Participants were ambivalent about receiving support messages during weekend drinking occasions, and stressed that, if they are used, they should be one way (ie, not interactive) to minimize burden and should focus on reminding individuals about their goals, only if they set one. Finally, participants were generally supportive of a Web-based dashboard incorporating features such as graphical feedback, gamification, social networking. This is not surprising given the recent growth in commercial programs to track health that utilize these online features.

Our findings complement existing literature describing formative research on how to design text message interventions for hazardous alcohol use prevention. For example, Sharpe et al [17] found that 18-60-year-olds with past hazardous alcohol use wanted text message content that was engaging, relevant, and useful for recipients, including reducing the complexity of message content and structure, increasing the interactivity, ensuring an empowering tone to text messages, and optimizing the appropriateness and relevance of text messages. Bock et al [18] found that community college students wanted messages that apply to specific drinking contexts, including messages for before and after a drinking occasion and messages that are tailored to different drinking habits (ie, age and drinking experience). In other work, Muench et al [19,20] found that individuals in addictions treatment programs tended to prefer benefit-driven over consequence-driven messages, and messages that are tailored to commonly encountered hypothetical situations. Our study is the first to have individuals comment on how they actually experienced the text message intervention in the real world and reflect on how the various features potentially impacted their thoughts, beliefs, and behaviors.

### Limitations

Findings may not be applicable to other populations, such as young adults with less severe alcohol use, or other age groups, such as adolescents. This is especially relevant given the high rates of comorbid tobacco and marijuana use in our cohort. We only recruited participants in the TRAC intervention arm who had completed at least 6 weeks (50%) of the SMS text messaging assessments, which could have resulted in undersampling the opinions of those who dropped out. Focus groups were conducted at least 6 months after completion of the TRAC intervention, and therefore recall of real-time perceptions might be influenced by memory biases. Finally, the users' opinions were based on their experiences with one intervention design, and may not be the same with different text message alcohol intervention designs.

### Conclusions

Young adults perceived the TRAC intervention as a useful way to help them reduce heavy drinking on weekends. Important themes regarding usability of the TRAC intervention included its ease of use, confidentiality, and its ability to increase personal awareness of alcohol consumption and accountability for personal drinking behavior. Focus group discussion indicated that text message interventions should attempt to personalize materials based on user-specific features, such as drinking severity, and provide additional support through online adjuncts.

## Appendix

Multimedia Appendix 1 The flow of text message branching logic for the Texting to Reduce Alcohol Consumption (TRAC) intervention.

## Abbreviations

AUDIT-C: Alcohol Use Disorders Identification Test Consumption
ED: emergency department
NIAAA: National Institute on Alcohol Abuse and Alcoholism
RCT: randomized controlled trial
SMS: short message service
TRAC: Texting to Reduce Alcohol Consumption
