# Supplementary figures and images for: Optimizing a Text Message Intervention to Reduce Heavy Drinking in Young Adults: Focus Group Findings
Source: JMIR Mhealth Uhealth. 2016 Jun 22;4(2):e73. doi: 10.2196/mhealth.5330 (PMC4935795; doi:10.2196/mhealth.5330)

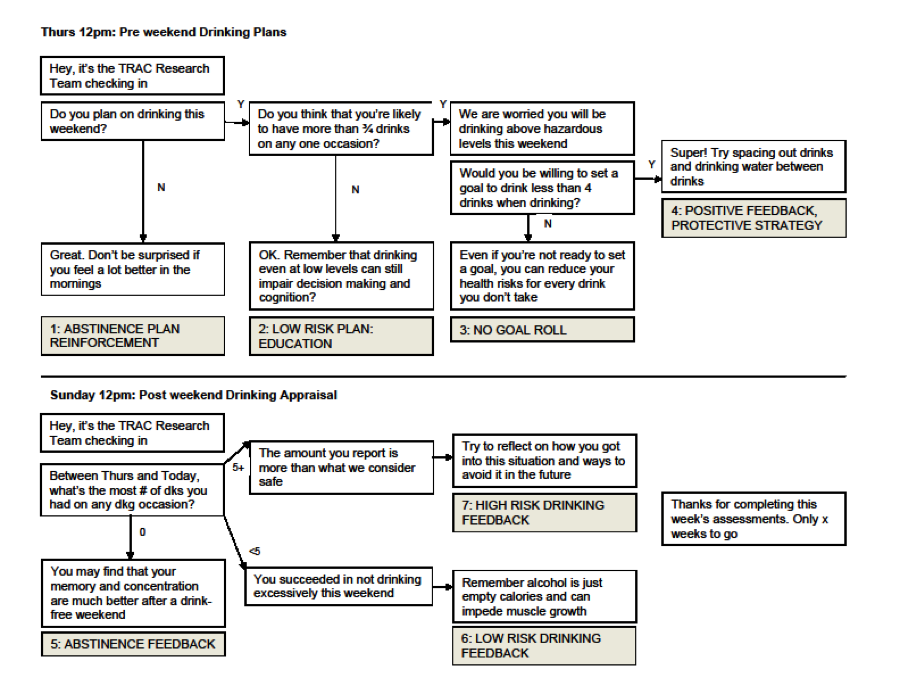

Supplement: Multimedia Appendix 1 [file mhealth_v4i2e73_app1.png]
